# Targeting Nox4 alleviates TMJ osteoarthritis by restoring redox homeostasis and suppressing ferroptosis in macrophages

**DOI:** 10.3389/fphar.2026.1854785

**Published:** 2026-08-06

**Authors:** Yucheng Wang, Qilin Li, Weijia Zhang, Rui Chao, Xuzhuo Chen, Yexin Wang, Shanyong Zhang, Jinze Zhen

**Affiliations:** 1 Department of Orthodontics, Qingdao University Affiliated Hospital, Qingdao, China; 2 Department of Oral Surgery, Shanghai Key Laboratory of Stomatology & Shanghai Research Institute of Stomatology, National Clinical Research Center for Oral Diseases, Shanghai Ninth People’s Hospital, College of Stomatology, Shanghai Jiao Tong University School of Medicine, Shanghai, China; 3 Pacific State Medical University Russia, Vladivostok, Russia; 4 Department of Oral Surgery, Fengcheng Hospital of Fengxian District, Shanghai Ninth People’s Hospital Fengcheng Branch Hospital, Shanghai, China

**Keywords:** ferroptosis, macrophage, NOX4, oxidative stress, TMJ OA

## Abstract

**Objectives:**

Temporomandibular osteoarthritis (TMJ OA) is a degenerative disease with pain, abnormal sound, and restricted mouth - opening. Oxidative stress and ferroptosis contribute to its progression, and inflammatory macrophages in the synovium are crucial. Nicotinamide adenine dinucleotide phosphate (NADPH) oxidase 4 (Nox4), a major Reactive Oxygen Species (ROS) source, is involved in joint disorders, but the mechanism of Nox4 regulating macrophage ferroptosis in TMJ OA is unclear.

**Method:**

RNA-sequencing was performed on synovial tissues obtained from patients or rats with or without TMJ OA to identify potential therapeutic targets for TMJ OA. Synovial tissues were examined histologically after staining with immunofluorescence. The impact of silencing Nox4 gene expression on inflammatory macrophages was explored. RT-qPCR and Western blotting were used to evaluate the expression of inflammatory and antioxidant genes. Intracellular and mitochondrial ROS were detected using DCFH-DA, DHE, or MitoSOX probes. Mitochondrial membrane potential was detected using the JC-1 probe. Lipid peroxidation and ferroptosis were measured using C11-BODIPY and FerroOrange. RNA-seq was used to analyze the anti-inflammatory mechanisms of the Nox4 inhibitor GLX351322 (40 μM) in LPS-stimulated macrophages.

**Results:**

In this study, we observed a marked upregulation of Nox4 expression in synovial macrophages from both TMJ OA patients and rats. Inhibition of Nox4 significantly attenuated inflammatory responses through activation of the nuclear factor erythroid 2-related factor 2 (Nrf2) pathway. Upon inhibition of Nox4, ROS production was reduced, accompanied by a reduction in mitochondrial damage. Meanwhile, Nox4 inhibition decreased ferroptosis in inflammatory macrophages by mitigating oxidative stress-induced lipid peroxidation.

**Conclusion:**

In this study, we identified Nox4 as a potential therapeutic target in TMJ OA. Targeting Nox4 counld reduce macrophage inflammation by alleviating oxidative stess-induced ferroptosis.

## 1 Introduction

Osteoarthritis (OA) is one of the most common degenerative diseases affecting the temporomandibular joint (TMJ), characterized by pain, abnormal sound, and restricted mouth opening, significantly impairing quality of life for patients (Cardone et al., 2022; Wang et al., 2015). Due to the complex pathogenesis of TMJ OA and scarcity of therapeutic interventions, it is extremely important to deeply explore the molecular mechanism of TMJ OA and find effective therapeutic targets. Accumulating evidence has highlighted the pivotal role of synovial macrophages inflammation in the progression of TMJ OA (Zhang et al., 2020; Cutolo et al., 2022). Notably, inflammatory macrophages, as a primary cellular component within the synovial tissue of TMJ, exacerbate articular cartilage and subchondral bone destruction by secreting abundant pro-inflammatory cytokines, such as tumor necrosis factor-α (Tnf-α), interleukin (IL) -1β, IL-6, and inducible nitric oxide synthase (iNOS) (Chen et al., 2019). Consequently, elucidating the molecular mechanisms underlying synovial macrophage inflammation presents a promising avenue for preventing and treating TMJ OA.

Accumulating evidence suggests that ferroptosis was a critical factor that contributes to the progression of TMJ OA (Jiang et al., 2020; Gong et al., 2023). Ferroptosis, a type of programmed cell death, results from the accumulation of cellular Reactive Oxygen Species (ROS) exceeding the redox capacity maintained by glutathione (GSH) and phospholipid hydroperoxide glutathione peroxidase (GPX4) (Gao and Jiang, 2018; Ingold et al., 2018). The accumulation of iron and lipid peroxides accelerates membrane lipid oxidation, leading to damage to the lipid bilayer of the plasma membrane (Stockwell et al., 2017; Stockwell and Jiang, 2020). Under inflammatory stimuli, macrophages exhibit enhanced phagocytic activity, resulting in intracellular iron accumulation (Zhang et al., 2012). Meanwhile, inflammatory cytokines secreted by inflammatory macrophages can exacerbate the progression of ferroptosis by modulating iron metabolism, lipid metabolism, and oxidative stress (Yang et al., 2022). Oxidative stress is characterized by excessive production of ROS, accompanied by mitochondrial dysfunction (Wang et al., 2021). Mitochondrial damage has also been implicated in the processes of lipid peroxidation and iron homeostasis disruption in bone metabolic diseases such as osteoporosis and osteoarthritis (Conrad et al., 2018). Furthermore, the excessive generation of ROS and activation of lipid peroxidation are closely linked to ferroptosis-dependent cell death processes (Stockwell et al., 2017; Stockwell and Jiang, 2020). Therefore, ferroptosis is not merely a consequence of inflammation but a dynamic process intricately intertwined with inflammatory progression. Therefore, inhibition of ferroptosis via alleviating oxidative stress-induced lipid hydroperoxides can aid in the development of novel therapeutic strategies against OA.

Nox4 is a constitutive enzyme that is widely expressed in organs and catalyzes the conversion of molecular oxygen into superoxide anions (^•^O_2_^−^) and ROS (Ogboo et al., 2022; Lambeth et al., 2007), exerting a pivotal role in the modulation of oxidative stress and downstream signals (Liao et al., 2022; Wang et al., 2022; Park et al., 2021). Growing evidence has shown that Nox4 plays a significant part in the pathogenesis of bone-related diseases (Lin et al., 2024). Recent research has unveiled that melatonin can inhibit the upregulation of Nox4 in mitochondria, thereby alleviating mitochondrial dysfunction, effectively suppressing ferroptosis, and ameliorate the symptoms of OA (Wang et al., 2024). Our previous study demonstrated that nanoparticles loaded with Nox4 inhibitor (GLX351322) effectively suppressed TBHP-induced inflammation, ROS production, and ferroptosis (Zhen et al., 2024). This is the first report on the role of Nox4 in TMJOA synovitis. Animal experiments were also conducted to further confirm the *in vivo* and *in vitro* effects of Nox4 on TMJOA synovitis. However, the mechanisms of how Nox4 regulates ferroptosis of inflammatory macrophages in TMJ OA remains unclear.

**SCHEME 1:**
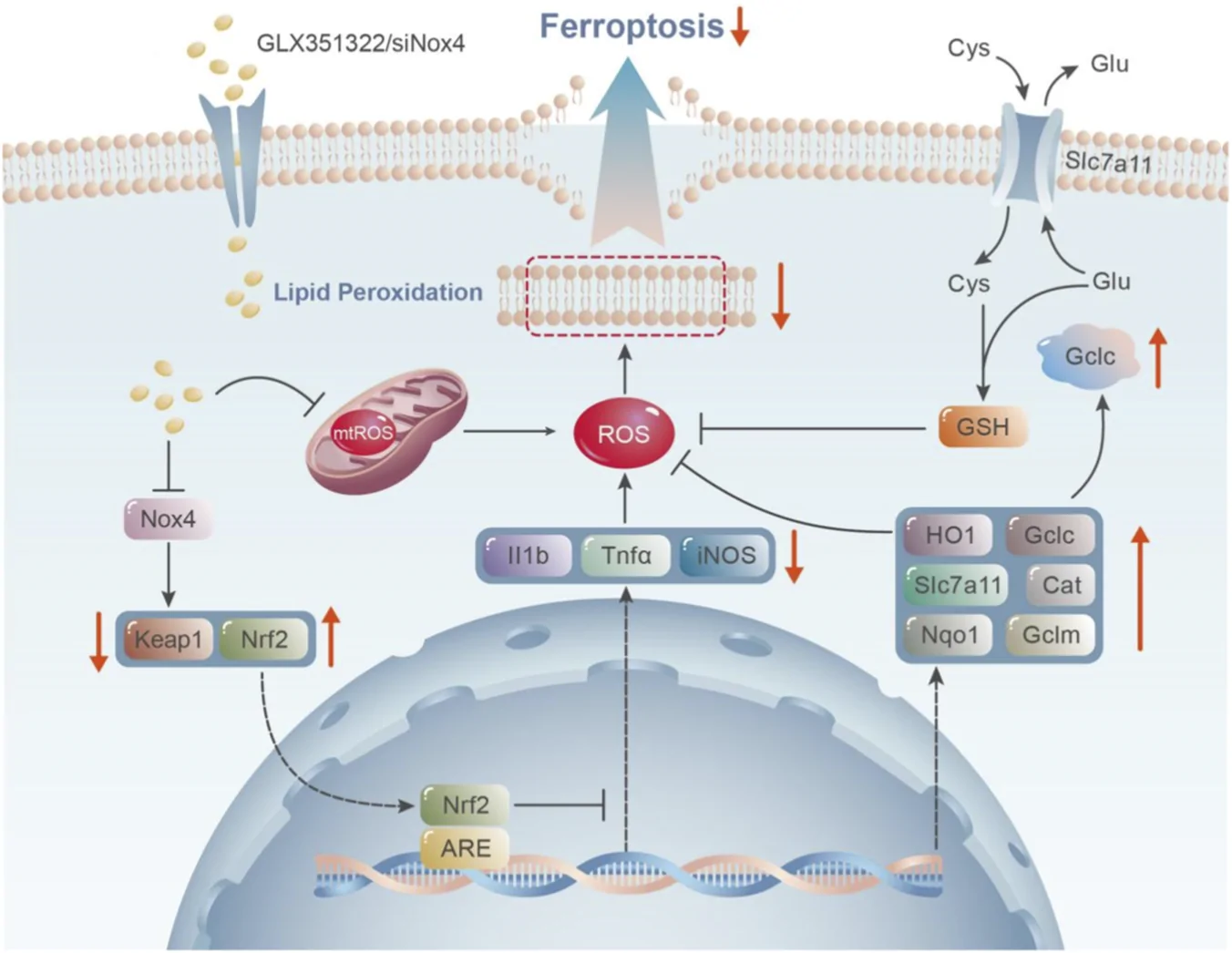
Schematic diagram of the protective effects of Nox4 inhibition against macrophage inflammation.

In our study, we demonstrated that Nox4 silencing and Nox4 inhibition by GLX351322 significantly ameliorated the inflammatory response in macrophages. Mechanistically, the inhibition of Nox4 may activate the Nrf2 signal pathway to eliminate intracellular ROS, protect against mitochondrial injury and alleviate ferroptosis via reduction of oxidative stress-induced lipid peroxidation. Our findings suggest that Nox4 may act as a prospective therapeutic target for TMJ OA (Scheme 1).

## 2 Materials and methods

### 2.1 Reagents and antibodies

GLX351322, an inhibitor of Nox4, was purchased from Selleck (Houston, TX, United States). Bacterial lipopolysaccharide (LPS) from *Escherichia coli* O55:B5 was purchased from InvivoGen (San Diego, CA, United States). Minimal essential medium alpha (α-MEM) was obtained from HyClone (Logan, UT, United States). Fetal bovine serum (FBS) was purchased from Avantor (Gaithersburg, MD, United States). Penicillin was purchased from Gibco BRL (Grand Island, NY, United States). HiScript III 1st Strand cDNA Synthesis Kit (+gDNA wiper) and ChamQ Universal SYBR qPCR Master Mix were obtained from Vazyme (Nanjing, China). DCFH-DA assay kit, Dihydroethidium (DHE) assay kit, Mitochondrial membrane potential assay kit (JC-1), and 2-(4-Amidinophenyl)-6-indolecarbamidine dihydrochloride (DAPI) were obtained from Beyotime Institute of Biotechnology (Shanghai, China). MitoSOX Red probe were purchased from Yeasen (Shanghai, China). Primary antibodies against Gapdh (CST #5174), Keap1 (CST #4678) and secondary antibodies, were purchased from Cell Signaling Technology (CST, Danvers, MA, United States). Primary antibodies against Nrf2 (Affinity #AF0639), HO-1 (Affinity #AF5393), Nqo1 (Affinity #DF6437) and Nox4 (Affinity #DF6924), were purchased from Affinity Biosciences (Cincinnati, OH, United States). Primary antibody against iNOS (Thermo Fisher #PA1-036) was purchased from Thermo Fisher Scientific (Waltham, MA, United States). Nox4 siRNA (siNox4) and negative control (NC) siRNA (siNC) were purchased from RiboBio (Guangzhou, China).

### 2.2 Clinical patient samples

Synovial tissue specimens of TMJ were collected from patients at the Ninth People’s Hospital affiliated to Shanghai Jiao Tong University School of Medicine. Comprehensive pathological examination was conducted to ensure the integrity of the samples. Some samples were immediately frozen in liquid nitrogen for subsequent RNA extraction and RNA-seq. Alternatively, some samples were fixed using 4% paraformaldehyde (PFA) to maintain tissue morphology and antigenicity for subsequent immunofluorescence staining experiments. This study was approved by the Ethics Committee of Shanghai Ninth People’s Hospital, Shanghai Jiao Tong University School of Medicine (No.SH9H-2022-T43-2). All patients were informed in detail about the study and provided their written informed consent.

### 2.3 RNA sequencing

To explore the differentially expressed genes (DEGs) between normal and inflammatory synovial tissues of the TMJ, six synovial samples were obtained from patients with TMJ OA and TMJ disk anterior displacement. These samples were immediately frozen in liquid nitrogen and stored until RNA extraction. Total RNA was isolated using Trizol reagent. Sequencing libraries were constructed with the NEBNext® Ultra™ RNA Library Prep Kit for Illumina® (#E7530L, NEB, United States), and sequencing was performed on an Illumina platform, generating 150 bp paired-end reads. The sequencing reads were aligned to the mm9 or hg19 reference genome using Hisat2. Differential gene expression analysis was conducted using the DESeq2 package, with significance defined as a Pvalue <0.05 and a log2 fold change < −1. Additionally, Gene Ontology (GO) enrichment analysis and signaling pathway enrichment analysis were performed using Metascape (www.metascape.org). Gene Set Enrichment Analysis (GSEA) was performed according to the developer’s guidelines (https://www.broadinstitute.org/gsea/).

### 2.4 Microarray sequencing

To explore the differential gene expression profiles between normal and inflamed synovial tissues of the TMJ, Six 8-week-old male Sprague-Dawley rats were allocated into two groups (n = 3): a control group and a CFA-induced inflammation group. The latter group underwent intra-articular injection of 50 μL of a 5 mg/mL solution of complete Freund’s adjuvant (CFA). Following a 3-day period, the rats were euthanized, and synovial tissues were harvested for total RNA extraction utilizing Trizol reagent. Subsequently, mRNA microarray analysis was conducted by KangChen Bio-tech (Shanghai, China) to investigate the gene expression patterns. Animal ethics was approved by the Institutional Animal Ethics Review Board of the Shanghai Ninth People’s Hospital, Shanghai Jiao Tong University School of Medicine (Approval No. SH9H-2020-A180-1). These sequencing data of animal tissue were originally reported in our published study (Zhen et al., 2023).

### 2.5 Cell culture

The RAW 264.7 murine macrophage cell line was kindly donated by the Cell Bank/Stem Cell Repository of the Chinese Academy of Sciences. These cells were cultured in complete α-MEM medium supplemented with 5% FBS, 100 units/mL of penicillin, and streptomycin. The incubation was conducted at 37 °C in an incubator with a 5% CO_2_ atmosphere. The cells were seeded once they reached a confluence of 70%–80%.

### 2.6 siRNA

To perform cell transfection, seed 1 × 10^5^ RAW 264.7 cells into each well of a 24-well plate containing aMEM medium supplemented with 5% FBS, 100 units/mL of penicillin, and streptomycin, ensuring a cell density of 30%–50% at the time of transfection. Initially, 1.25 μL of 20 μM siRNA stock solution was diluted in 30 μL of 1X riboFECT™ CP Buffer. Next, 3 μL of 1X riboFECT™ CP Reagent was added, and the mixture was gently pipetted to ensure homogeneity, followed by incubation at room temperature for 0–15 min to form the transfection complex. Subsequently, the riboFECT™ CP transfection complex was added to complete medium without antibiotics. The culture plate was then placed in a 37 °C CO_2_ incubator for 48 h, followed by exposure to LPS stimulation for 24 h. The details of four transfection groups including NC, NC + LPS, siNox4, and siNox4+LPS. Each transfection sample was set up with at least 3 replicate wells.

### 2.7 Quantitative PCR analysis

To evaluate the expression of anti-inflammatory and antioxidant-related genes, RAW 264.7 were seeded in 6-well plates at a density of 4 × 10^5^ cells/well, after 48 h siRNA transfection, cultured with or without LPS for another 1 day. Total RNA was isolated using Trizol reagent, following the manufacturer’s protocol. Subsequently, 0.5 mg of the purified RNA was used to reverse transcription (RT) utilizing the HiScript III 1st Strand cDNA Synthesis Kit (R312-01; Vazyme, China). For quantitative RT-PCR, the ChamQ Universal SYBR qPCR Master Mix (Q711; Vazyme, China) was employed. Expression levels were normalized to GAPDH abundance through application of the 2^−ΔΔCT^ method. The standard deviation (S.D.) of technical replicates is indicated by error bars. The primer sequences utilized in this study are listed in Table 1.

**TABLE 1:** Sequences of the RT-qPCR.

Gene	Forward	Reverse
Nox4	TGC​CTG​CTC​ATT​TGG​CTG​T	CCG​GCA​CAT​AGG​TAA​AAG​GAT​G
Il1β	TCG​CAG​CAG​CAC​ATC​AAC​AAG​AG	AGG​TCC​ACG​GGA​AAG​ACA​CAG​G
iNOS	ACT​CAG​CCA​AGC​CCT​CAC​CTA​C	TCC​AAT​CTC​TGC​CTA​TCC​GTC​TCG
TNF-α	CAG​GCG​GTG​CCT​ATG​TCT​C	CGA​TCA​CCC​CGA​AGT​TCA​GTA​G
Hmox1	GAG​CAG​AAC​CAG​CCT​GAA​CT	AAA​TCC​TGG​GGC​ATG​CTG​TC
Cat	GGA​GGC​GGG​AAC​CCA​ATA​G	GTG​TGC​CAT​CTC​GTC​AGT​GAA
Keap1	CGG​GGA​CGC​AGT​GAT​GTA​TG	TGT​GTA​GCT​GAA​GGT​TCG​GTT​A
Nrf2	CTT​TAG​TCA​GCG​ACA​GAA​GGA​C	AGG​CAT​CTT​GTT​TGG​GAA​TGT​G
GAPDH	GGT​GAA​GGT​CGG​TGT​GAA​CG	GGT​GAA​GGT​CGG​TGT​GAA​CG

### 2.8 Western blotting

To evaluate the expression of anti-inflammatory and antioxidant-related proteins, RAW 264.7 cells were seeded in 6-well plates at a density of 5 × 10^5^ cells/well, after 48 h siRNA transfection, cultured with or without LPS for another 1 day. Total protein was extracted using a buffer solution containing protease inhibitors (P8340; Sigma-Aldrich, China) to release the proteins. Following extraction, cellular debris was removed by centrifugation, and the supernatant was collected as the protein sample. The protein concentration was determined using a Bicinchoninic acid (BCA) protein assay kit (23225; Thermo Fisher Scientific, United States). Samples were mixed with BCA reagent, and the absorbance was measured at a specific wavelength to calculate the protein concentration. Subsequently, the protein samples were dissolved in a loading buffer and separated by 10% SDS-PAGE gel electrophoresis. The proteins on the SDS-PAGE gel were then transferred onto a 0.22 μm polyvinylidene fluoride (PVDF) membrane. The membrane was blocked with 5% BSA in 1 × TBST for 1 h at room temperature. Afterward, it was incubated overnight at 4 °C with primary antibodies (Nox4, 1:1,000; Keap1, 1:1,000; Nrf2, 1:1,000; Hmox1, 1:1,000; Nqo1, 1:1,000; Gapdh, 1:1,000). Following incubation with fluorescent secondary antibodies for 1 h at room temperature, the blots were visualized using the Odyssey V3.0 image scanner (Li-COR Inc., Lincoln, NE, United States).

### 2.9 Immunofluorescence staining

Cells were fixed with 4% PFA for 30 min, followed by an overnight incubation at 4 °C with the primary antibody against iNOS (#PA1-036; Thermo Fisher, United States). Subsequently, the secondary antibody was incubated for 1 h at room temperature. The secondary antibodies for immunofluorescence were incubated for another 1 h. DAPI was used for nuclei staining, and the cells were visualized using confocal laser scanning microscopy (CLSM). The relative intensity of fluorescence was quantitatively analyzed utilizing ImageJ software.

For immunofluorescence analysis of synovium samples derived from temporomandibular joints, the tissues were embedded in paraffin at a predefined angle and processed into histological sections. These sections were then stained with antibodies against Nox4 (#DF6924; Affinity, United States). Examination and photograph of the stained sections were carried out using a high-resolution Leica DM4000B microscope. The proportion of positive cells was quantitated with the assistance of ImageJ software.

### 2.10 Intracellular ROS detection

Intracellular levels of ROS were measured employing the DCFH-DA probe (S0033S; Beyotime Biotechnology, China) and the DHE probe (HY-D0079; MedChem Express, China). RAW 264.7 macrophages were plated and subjected to a 48-h siRNA transfection, followed by culturing with or without LPS. Subsequently, the cells were incubated in serum-free medium containing 10 μM DCFH-DA for 20 min at 37 °C, or stained with 5 μM DHE probe for 30 min. After that, the cells were stained with either Hoechst 33,342 or DAPI for nuclear fluorescence, then washed 3 times with warm phosphate-buffered saline (PBS) to remove excess dye. Fluorescent images were captured via CLSM, and semi-quantitative assessment of ROS-positive cells was performed using the ImageJ software.

### 2.11 Intracellular mitochondrial membrane potential assay

Mitochondrial membrane potential was assessed utilizing a JC-1 Mitochondrial membrane potential assay kit (C2006; Beyotime Biotechnology, China). RAW 264.7 macrophages were plated and subjected to a 48-h siRNA transfection, followed by culturing with or without LPS for 24 h. Then, the cells were incubated with the JC-1 working solution at 37 °C for 20 min, washed 3 times with the JC-1 staining buffer. Observations were made using CLSM. Semi-quantitative analysis of the JC-1 aggregates/monomer fluorescence ratio was conducted using ImageJ software.

### 2.12 Mitochondrial ROS detection

Mitochondrial ROS generation was assessed using MitoSox Red probe (Yeasen, China; 40778ES50). RAW 264.7 macrophages were plated and subjected to a 48-h siRNA transfection, followed by culturing with or without LPS for 24 h. Then, the cells were incubated with serum-free medium containing 5 μM MitoSox Red probe for 10 min at 37 °C. Subsequently, the cell nuclei were stained with Hoechst 33,342. The cells were washed three times with warm PBS to remove excess dye, and visualized using CLSM.

### 2.13 Detection of lipid peroxidation

Lipid peroxidation was detected using the C11-BODIPY 581/591 fluorescent probe (D3861; Invitrogen, Carlsbad, CA, United States). RAW 264.7 macrophages were plated and subjected to a 48-h siRNA transfection, followed by culturing with or without LPS for 24 h, RAW 264.7 cells were washed with PBS and incubated with 5 μM C11-BODIPY working solution at 37 °C for 1 h. Cells were then rinsed three times with PBS to remove the excess dye. Fluorescent images were acquired using CLSM. The fluorescence intensity of oxidized (green) and non-oxidized (red) forms was semiquantitative analyzed using ImageJ software.

### 2.14 Detection of intracellular ferrous iron

Intracellular ferrous iron (Fe^2+^) levels were detected using the FerroOrange fluorescent probe (F374; Dojindo, Shanghai, China). RAW 264.7 macrophages were plated and subjected to a 48-h siRNA transfection, followed by culturing with or without LPS for 24 h, RAW 264.7 cells were washed with PBS and incubated with 1 μM FerroOrange working solution at 37 °C for 30 min in the dark. After three washes with PBS, fluorescence images were captured by CLSM. The mean fluorescence intensity was semiquantitative analyzed using ImageJ software.

### 2.15 Statistical analysis

The data were analyzed using GraphPad Prism 9 software (GraphPad Software, San Diego, CA, United States) and presented as mean ± SD. Experiments were conducted independently repeated three times. One-way analysis of variance (ANOVA) was employed for comparing multiple experimental groups, with a P-value of <0.05 considered significant. Significance levels were indicated as follows: * for *P* < 0.05, ** for *P* < 0.01, *** for *P* < 0.001, and NS for not significant.

## 3 Results and discussion

### 3.1 RNA-seq analysis revealed upregulation of Nox4 in the synovium of TMJ OA in humans and rats

**FIGURE 1:**
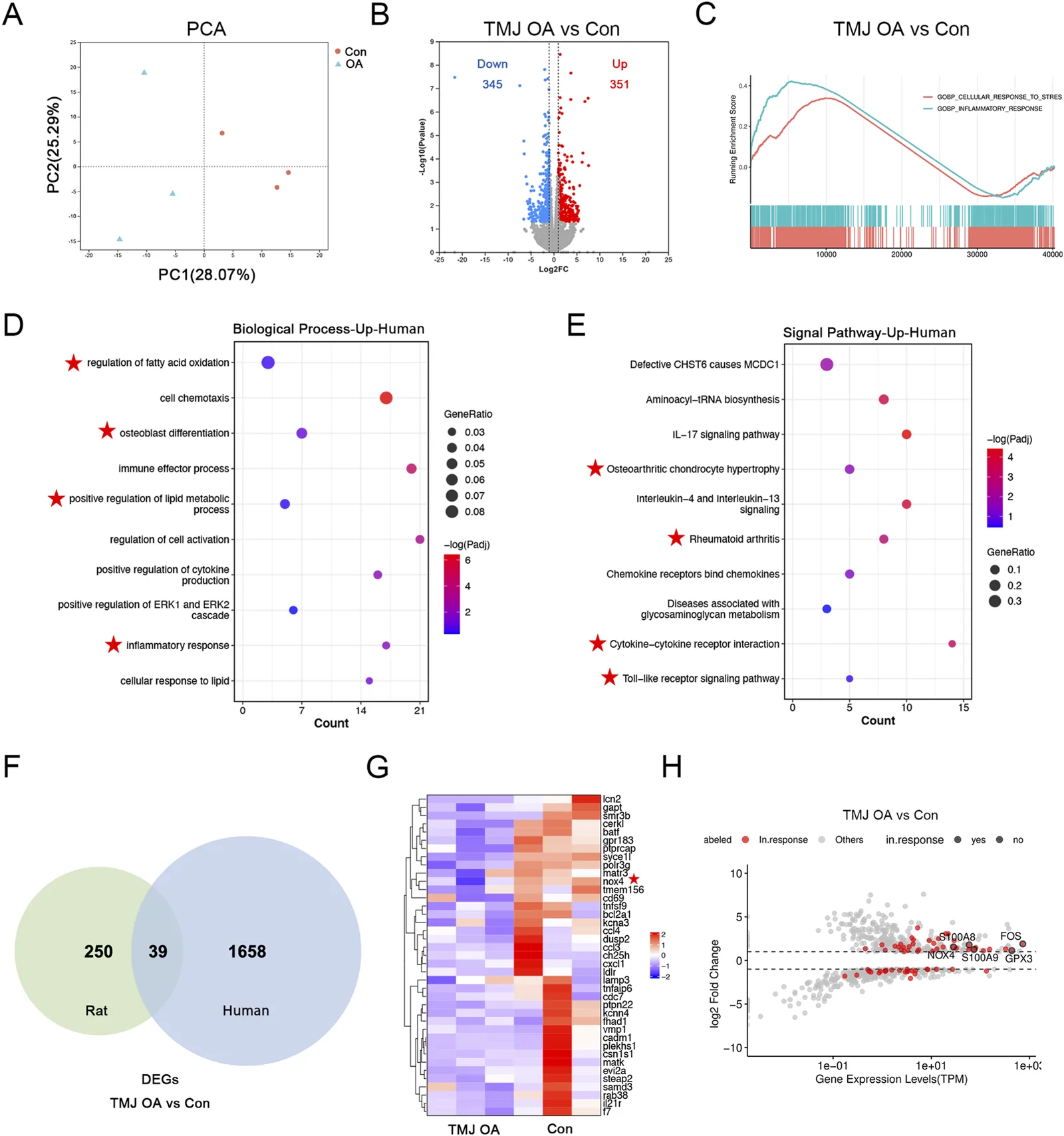
RNA-seq analysis revealed upregulation of Nox4 in the synovium of TMJ OA in humans and rats. **(A)** PCA of the different transcriptomic profiles among the Control and TMJ OA group. **(B)** Volcano plots of differentially regulated genes between the Control and TMJ OA group. **(C)** GSEA of the expressed genes between the Control and TMJ OA group. **(D)** Bubbleplot of biological process enrichment analysis of differentially upregulated genes (TMJ OA vs. Control, Human). **(E)** Bubbleplot of signaling pathway enrichment analysis of differentially downregulated genes (TMJ OA vs. Control, Human). **(F)** Bubbleplot of biological process enrichment analysis of differentially downregulated genes (TMJ OA vs. Control, Human) **(G)** Bubbleplot of biological process enrichment analysis of differentially upregulated genes (TMJ OA vs. Control, Rat). **(H)** Venn diagram of differentially upregulated genes (Log2FC ≥ 1, Pvalue<0.05). **(I)** Heatmap of 39 differentially upregulated genes identified in the Venn diagram **(H)**. **(J)** Volcano plots of Nox4 expression.

To investigate the potential targets in TMJ OA, we conducted RNA sequencing on synovial tissues from patients with different conditions, including three cases of TMJ OA and three controls (Con) with temporomandibular joint disk anterior displacement. Principal components analysis (PCA) of the transcriptomic data revealed distinct transcriptomic profiles between the Con and TMJ OA groups (Figure 1A). The volcano plot showed significant upregulation of 351 genes and downregulation of 345 genes in the synovium of patients with TMJ OA (Figure 1B). GSEA revealed that genes involved in cellular stress response and inflammatory response were prominently enriched, indicating a significant role in regulating oxidative stress in TMJ OA (Figure 1C). Moreover, GO enrichment analysis also showed that biological processes such as regulation of fatty acid oxidation, osteoclast differentiation, positive regulation of lipid metabolic processes, and inflammatory response were significantly associated with the pathogenesis of TMJ OA (Figure 1D). Pathway enrichment analysis revealed strong associations with Osteoarthritic chondrocyte hypertrophy, Rheumatoid arthritis, Cytokine-cytokine receptor interaction, and Toll-like receptor signaling pathways in the synovium of patients with TMJ OA (Figure 1E). Genes that were downregulated were associated with biological processes such as cell-cell adhesion and extracellular matrix organization (Figure 1F).

We induced TMJ inflammation in rats by intra-articular injecting CFA, subsequently harvested the synovial tissues for RNA-seq and conducted a combined analysis of differentially expressed genes in both humans and rats. Consistent with the differentially expressed genes (DEGs) previously found in human synovial tissues, GO enrichment analysis revealed a significant enrichment of biological processes, including the regulation of macrophage activation, inflammatory response, and positive regulation of cytokine production, within the synovium of rats exhibiting TMJ OA (Figure 1G). The Venn diagram displayed the overlapping upregulated DEGs in the synovium of both humans and rats, comparing TMJ OA with controls (log2FoldChange>2, Pvalue<0.05) (Figure 1H). Furthermore, the heatmap visualized the expression of 39 DEGs in the synovium of humans (Figure 1I) and revealed a significant upregulation of Nox4 expression, with approximately 2.92-fold increase in human TMJ OA samples and approximately 2.30-fold increase in the rat model. This finding was further supported by a scatter plot, which clearly demonstrated elevated Nox4 expression levels in the synovium of patients with TMJ OA (Figure 1J).

### 3.2 Silencing Nox4 gene expression reduced inflammatory response and activates Nrf2 signaling pathway in LPS-stimulated macrophages

**FIGURE 2:**
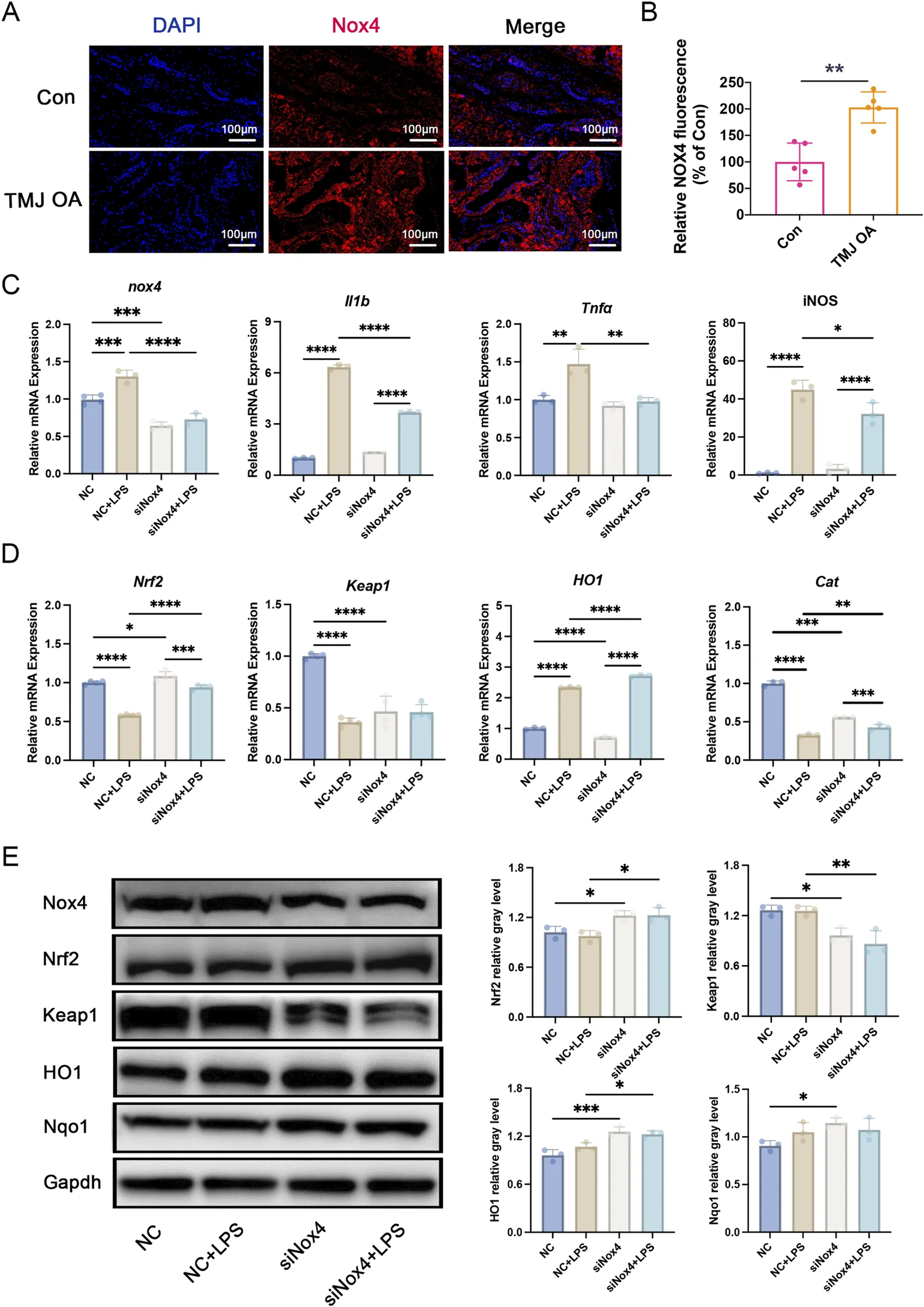
Silencing Nox4 gene expression reduced inflammatory response and activated Nrf2 signaling pathway in LPS-stimulated macrophages. **(A)** Immunofluorescence of Nox4 in synovium of patients with or without TMJ OA. **(B)** The percentage of Nox4 fluorescence. **(C)** RT-qPCR results of inflammatory genes expression. **(D)** RT-qPCR results of anti-oxidant genes expression. **(E)** Protein expression level of Nox4, Nrf2, Keap1, HO-1 and Nqo1.

After establishing the significance of Nox4 in the progression of TMJ OA in both humans and rats, we conducted an immunofluorescence analysis on synovial tissues obtained from patients with and without TMJ OA (Figure 2A). Our findings revealed a notable upregulation in Nox4 expression within the synovial tissues of the TMJ OA group, showing a significant increase in Nox4-associated fluorescence intensity when compared to the control group. Consequently, we sought to investigate whether the inhibition of Nox4 using siRNA could attenuate inflammatory responses in LPS-stimulated macrophages. RAW 264.7 macrophages were transfected with Nox4 siRNA. Nox4 expression was significantly upregulated after LPS stimulation and effectively decreased with siNox4 treatment (Figure 2C). LPS stimulation resulted in an upregulation of mRNA levels of pro-inflammatory genes such as Tnf-α, IL-6, and iNOS. However, treatment with siNox4 notably decreased these levels, confirming the anti-inflammatory effect of silencing Nox4 expression in inflammatory macrophages. To further verify whether the anti-inflammatory effect of Nox4 silencing is dependent on the Nrf2 signaling pathway, we performed rescue experiments using the specific Nrf2 inhibitor ML385 in LPS-activated macrophages. RT-qPCR results showed that ML385 treatment effectively reversed the inhibitory effect of siNox4 on the mRNA expression of pro-inflammatory genes (Tnf, Il1b, and Nos2), significantly restoring their expression levels (Supplementary Figure S1). These rescue data functionally validated that Nrf2 activation is required for the anti-inflammatory effect induced by Nox4 inhibition.

We next investigated the anti-inflammatory mechanism of Nox4 inhibition in inflammatory macrophages. As depicted in Figure 2D, RT-qPCR data revealed that Nrf2 expression was downregulated upon LPS stimulation but notably upregulated following Nox4 silencing. The expression of Keap1 was significantly suppressed when Nox4 was silenced, compared to the NC group. RT-qPCR showed the Nrf2 downstream antioxidant genes, such as HO-1 and CAT, were significantly upregulated in the siNox4+LPS group, compared to the LPS group. To gain further insights into the mechanisms by which Nox4 modulates endogenous antioxidant systems, we examined the protein expression of Nrf2 downstream antioxidant genes using Western blot. Western blot results revealed a notable decrease in Nox4 protein levels in both the siNox4 and siNox4+LPS groups, accompanied by a significant upregulation of Nrf2 and its downstream antioxidant enzymes, HO-1 and Nqo1. Conversely, a marked downregulation of Keap1 protein expression was observed in both the siNox4 and siNox4+LPS groups (Figure 2E).

### 3.3 Silencing Nox4 gene expression reduced intracellular ROS in LPS-stimulated macrophages

**FIGURE 3:**
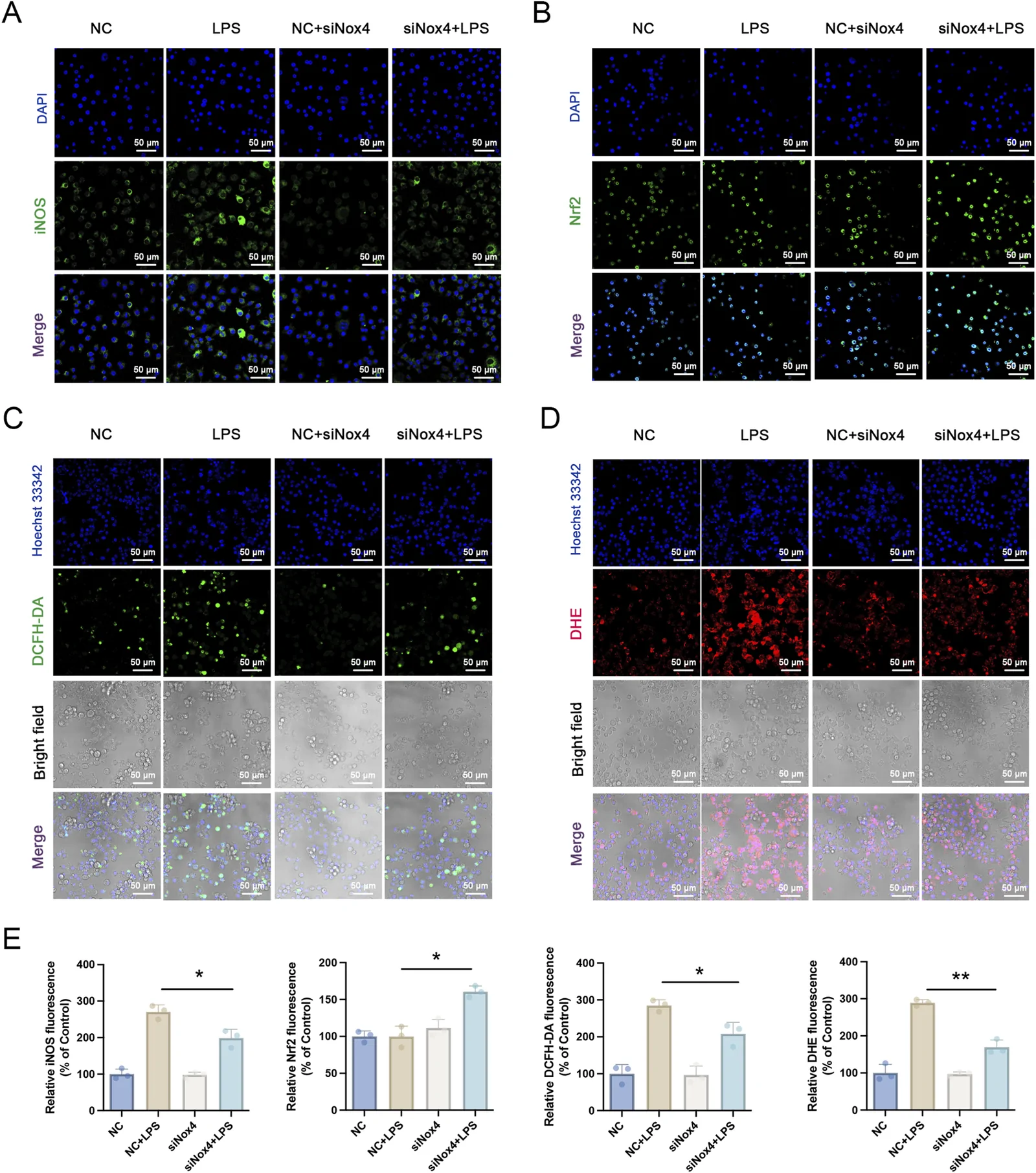
Silencing Nox4 gene expression reduced ROS in LPS-stimulated macrophage. **(A,B)** Respective confocal images of LPS-activated RAW 264.7 stained with iNOS and Nrf2. **(C,D)** Representative confocal images of DCFH-DA and DHE probes. **(E)** Representative quantitative analysis of relative fluorescence of iNOS, Nrf2, and ROS-positive cells.

After confirming that silencing Nox4 could inhibit inflammatory response via activating the endogenous antioxidant system. We next explored the effect of silencing Nox4 expression on inflammatory factors and intracellular ROS production. As demonstrated in Figure 3A, immunofluorescence staining revealed a significant upregulation of iNOS expression upon LPS stimulation, which was effectively suppressed following Nox4 silencing. Furthermore, Figure 3B illustrates that accumulation of Nrf2 was markedly enhanced after Nox4 silencing, indicating the activation of endogenous antioxidant systems in LPS-stimulated macrophages. To quantify the levels of oxidative stress, we utilized DCFH-DA, a well-established fluorescent probe for detecting intracellular ROS. As shown in Figure 3C, LPS stimulation led to a substantial increase in DCFH-DA fluorescence, indicating excessive ROS production in inflammatory macrophages. Conversely, the inhibition of Nox4 expression significantly decreased DCFH-DA fluorescence, suggesting a reduction in oxidative stress. Additionally, we assessed the intracellular concentration of ^•^O_2_^−^ using the DHE probe. As depicted in Figure 3D, LPS stimulation notably elevated DHE fluorescence, indicating excessive superoxide anion production. However, the suppression of Nox4 expression led to a significant decrease in ^•^O_2_^−^ production in inflammatory macrophages. To further validate the role of Nox4 in regulating oxidative stress, we performed reverse validation experiments by overexpressing Nox4. As shown in Supplementary Figures S2A, S2B, Nox4 overexpression significantly increased DCFH-DA fluorescence compared to LPS treatment alone. Our findings were validated by the fluorescence quantification of iNOS, Nrf2, DCFH-DA, and DHE (Figure 3E).

### 3.4 Transcriptomic analysis revealed the anti-inflammatory and antioxidant mechanisms of Nox4 inhibition

**FIGURE 4:**
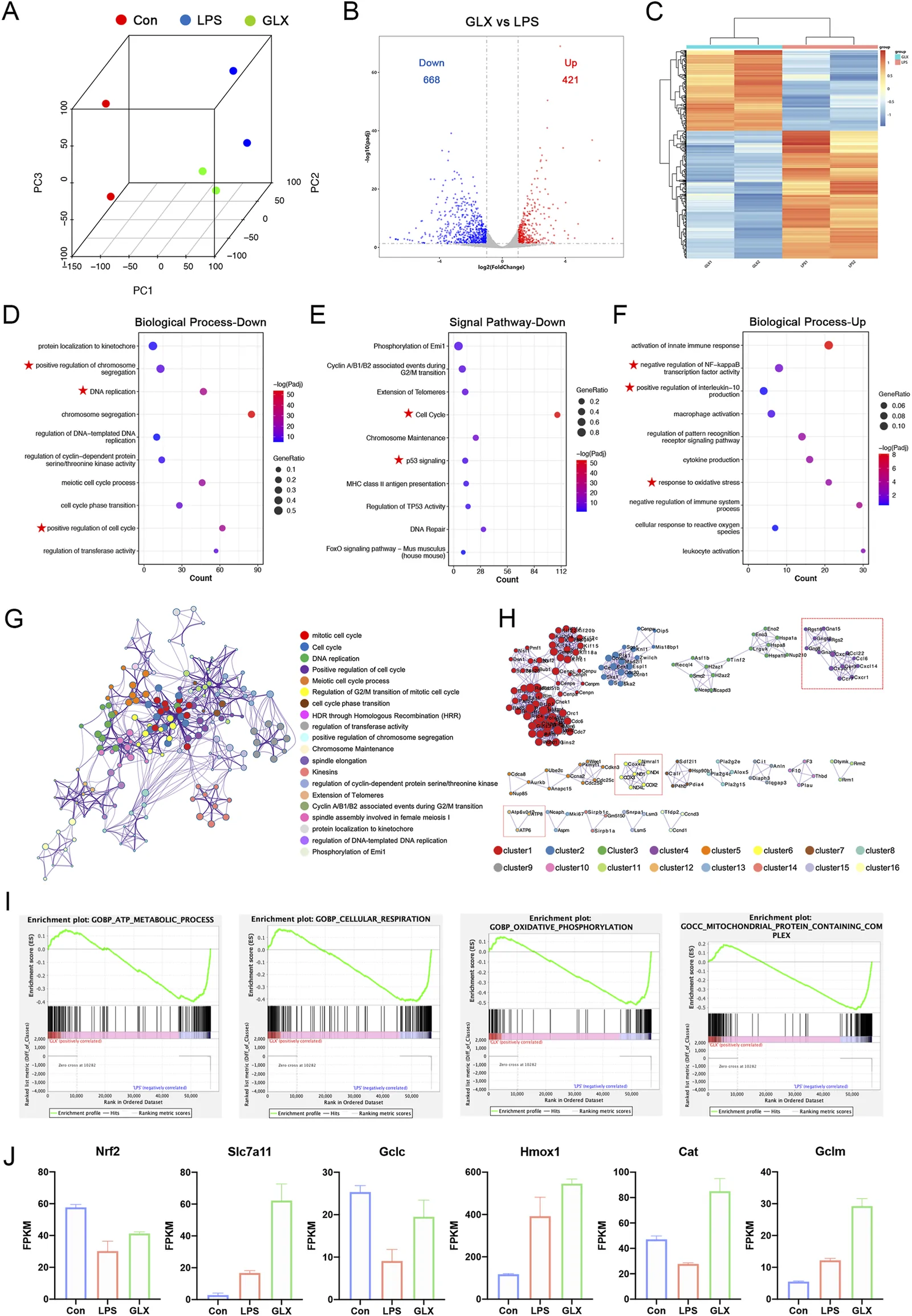
Transcriptomic analysis revealed the anti-inflammatory and antioxidant effect of Nox4 inhibition by GLX351322. **(A)** PCA of the different transcriptomic profiles among the Control, LPS and GLX group. **(B)** Volcano plots of differentially regulated genes between the LPS and GLX group. **(C)** Heatmap of differentially regulated genes between the LPS and GLX group. **(D)** Bubbleplot of biological process enrichment analysis of differentially downregulated genes (GLV vs. LPS). **(E)** Bubbleplot of signaling pathway enrichment analysis of differentially downregulated genes (GLV vs. LPS). **(F)** Bubbleplot of biological process enrichment analysis of differentially upregulated genes (GLV vs. LPS). **(G)** Biological process network of differentially downregulated genes (GLV vs. LPS). **(H)** Protein-protein interaction enrichment analysis of differentially downregulated genes (GLV vs. LPS). **(I)** GSEA of the expressed genes between the LPS and GLX group. **(J)** FPKM of Nrf2 downstream genes (Padj<0.05).

To gain further insights into the anti-inflammatory and antioxidant mechanisms of Nox4 inhibition, RNA sequencing was performed in LPS-activated macrophages treated with Nox4 inhibitor (GLX351322). PCA showed distinct transcriptomic profiles among the Con, LPS, and LPS + GLX351322 (GLX) groups (Figure 4A). Volcano plots demonstrated that 421 genes were significantly upregulated and 668 genes downregulated after treatment with GLX (Figure 4B). The heatmap displayed that treatment with GLX had a more pronounced suppressive effect on gene expression (Figure 4C). Therefore, we initially focused on the downregulated genes after GLX treatment (log2FC<-1, Padj<0.05). GO enrichment analysis was used to investigate the potential impacts of GLX on biological processes. As shown in Figure 4D, positive regulation of chromosome segregation, DNA replication, positive regulation of cell cycle, etc., were prominently related to the therapeutic mechanisms of GLX. Pathway enrichment analysis showed that Cell Cycle, p53 signaling were significantly enriched after GLX treatment (Figure 4E). Meanwhile, the GO enrichment analysis showed that negative regulation of NF-kappaB transcription factor activity, positive regulation of interleukin-10 production and response to oxidative stress were enriched in the upregulated genes after GLX treatment (Figure 4F), indicating the anti-inflammatory effect of GLX. Network of enriched terms showed that cell cycle related groups were enriched (Figure 4G). To further explore the potential protein-protein interactions affected by GLX, we performed protein-protein interaction enrichment analysis to identify interaction clusters. As depicted in Figure 4H, Cluster 4 was related to Chemokine signaling pathway, Cluster 6 was related to respiratory electron transport chain and Cluster 12 was related to Oxidative phosphorylation. In addition, GSEA revealed that genes related to ATP metabolic process, Cellular respiration, Oxidative phosphorylation and Mitochondrial protein containing complex were significantly enriched (Figure 4I). Additionally, as illustrated in Figure 4J, the expression of Nrf2 was significantly upregulated after GLX treatment, and its downstream genes, including Slc7a11, Gclc, Hmox1, Cat, and Gclm, were significantly upregulated. Collectively, Nox4 inhibition may exhibit anti-inflammatory and antioxidant effects by protecting mitochondria and alleviating ferroptosis.

### 3.5 Silencing Nox4 gene expression ameliorated ferroptosis by protecting against mitochondrial injury and lipid peroxidation

**FIGURE 5:**
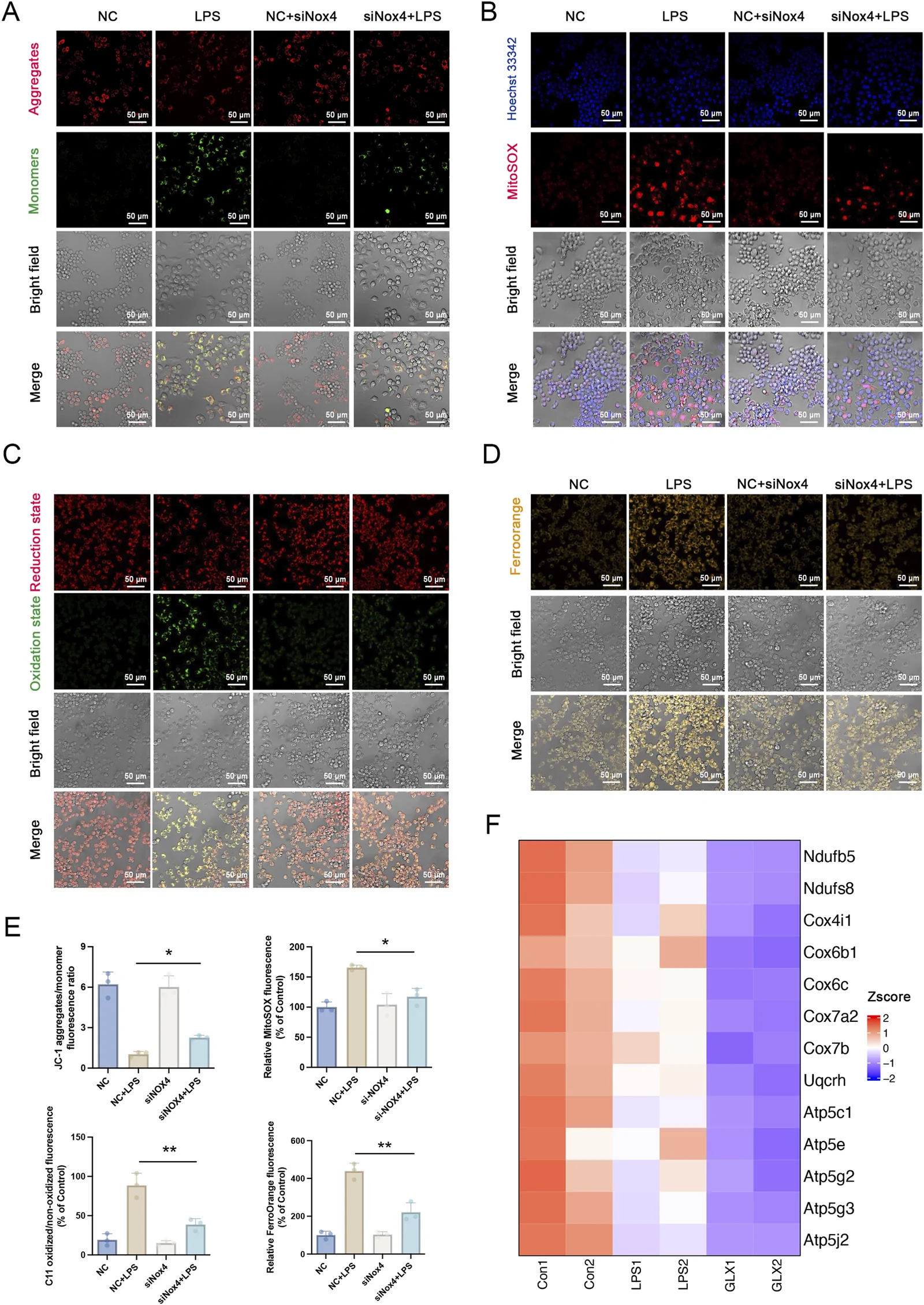
Silencing Nox4 gene expression ameliorated ferroptosis by protecting against mitochondrial injury and lipid peroxidation. **(A–D)** Respective confocal images of LPS-activated RAW 264.7 stained with JC-1, MitoSOX, C11-BODIPY, Ferroorange probe. **(E)** Quantitative analysis of relative fluorescence of **(A–D)**. **(F)** Heatmap of mitochondrial complex related genes.

Having demonstrated that Nox4 inhibition may protect against mitochondrial damage and ameliorates ferroptosis in inflammatory macrophages. To investigate the impact of silencing Nox4 expression on mitochondrial oxidative injury, we used the JC-1 mitochondrial membrane potential probe. The results showed that under LPS stimulation, the mitochondrial membrane potential was notably reduced, as evidenced by an increase in JC-1 monomers (green fluorescence). However, treatment with siNox4 effectively restored the mitochondrial membrane potential, as indicated by a decrease in JC-1 monomers (green fluorescence) and an increase in JC-1 aggregates (red fluorescence) (Figure 5A). Furthermore, the effect of siNox4 on mitochondrial ROS was assessed using MitoSOX staining. As depicted in Figure 5B, LPS stimulation significantly increased the fluorescence intensity of MitoSOX, indicating elevated mitochondrial ROS production during oxidative injury. However, silencing Nox4 reduced the level of mitochondrial ROS. Next, we investigated whether inhibiting Nox4 could alleviate ferroptosis by inhibiting lipid peroxidation. We used the C11-BODIPY probe to measure intracellular lipid peroxidation, a crucial indicator of oxidative stress. As depicted in Figure 5C, LPS stimulation markedly increased the lipid peroxidation level in RAW 264.7, as evidenced by enhanced green fluorescence (indicating oxidized state) and reduced red fluorescence (indicating non-oxidized state). However, silencing Nox4 significantly reduced the lipid peroxidation level, as indicated by increased red fluorescence (non-oxidized state) and decreased green fluorescence (oxidized state). Furthermore, we detected the level of ferroptosis using FerroOrange probe (Figure 5D). While these fluorescent probes provide reliable indicators, we further evaluated ferroptosis-related biochemical markers. As shown in Supplementary Figure S3A, silencing Nox4 significantly restored GPX4 protein expression, which was markedly reduced upon LPS stimulation. Additionally, LPS-induced depletion of intracellular GSH and reduction of the GSH/GSSG ratio were reversed by Nox4 silencing (Supplementary Figure S3B). Our findings were validated by the fluorescence quantification results of JC-1, MitoSOX, C11-BODIPY, and FerroOrange probes (Figure 5E). Reverse validation through Nox4 overexpression further supported its pro-ferroptotic role, as Nox4 overexpression significantly upregulated Acsl4 (Supplementary Figure S3C). Additionally, the expression levels of genes related to mitochondrial complexes were significantly decreased (Figure 5F). In summary, these findings highlight the capacity of Nox4 inhibition to suppress ROS generation by protecting mitochondria from oxidative stress-induced damage, thereby inhibiting lipid peroxidation and consequently ameliorating ferroptosis.

## 4 Discussion

Temporolmandibular osteoarthritis is characterized by synovitis, cartilage loss, and subchondral bone remodeling. However, the underlying mechanisms of TMJ OA remain largely unknown. To elucidate these mechanisms, RNA-sequencing was performed on synovial tissues obtained from patients or rats with or without TMJ OA to identify potential therapeutic targets. We conducted a combined analysis of differentially expressed genes that were significantly upregulated in both human and rat synovium and found a notable enrichment of Nox4 in the TMJ OA group compared to the control group. Specifically, a Venn diagram identified 39 overlapping upregulated genes between humans and rats (log_2_FC > 2, P < 0.05), and Nox4 was among the most upregulation genes. This upregulation was further validated by immunofluorescence staining in an expanded clinical cohort, confirming the clinical relevance of Nox4 in TMJ OA pathogenesis. Moreover, GSEA and GO enrichment analyses in human synovium revealed that biological processes such as regulation of fatty acid oxidation, osteoclast differentiation, and inflammatory response were prominently associated with TMJ OA, highlighting a significant role of oxidative stress in this disease.

Research has confirmed that in condylar chondrocytes, NOX4 is a key molecule responsible for extracellular matrix degradation and cartilage degeneration. In TMJOA, NOX4 activity is enhanced, leading to excessive production of ROS, which activates the downstream MAPK signaling pathway, increases the expression of inflammatory factors, and accelerates cartilage degradation. The use of a specific NOX4 inhibitor (e.g., GLX351322) effectively reduces ROS levels, suppresses catabolic responses, and thus protects chondrocytes (Lin et al., 2024). Importantly, our previously published studies have further demonstrated that GLX351322 significantly alleviates synovial inflammation and delays disease progression in a rat TMJ OA model, providing direct *in vivo* therapeutic evidence that supports NOX4 as a promising target for TMJ OA. Additionally, our previous study has shown that in synovial tissue, NOX4 is mainly expressed in macrophages. Under inflammatory stimulation (e.g., lipopolysaccharide, LPS), NOX4 expression in synovial macrophages is significantly upregulated, generating large amounts of ROS. This continuously activates the downstream MAPK and NF-κB signaling pathways, prompting macrophages to secrete abundant inflammatory cytokines, thereby triggering a robust synovial inflammatory response (Zhen et al., 2024).

Previous research has demonstrated that Nox4 augments the expression of inflammatory cytokines, thereby driving macrophage polarization towards an inflammatory phenotype (Li J. et al., 2023). Therefore, we sought to investigate the anti-inflammatory effect of silencing Nox4 in LPS-stimulated macrophages. Consistently, our results showed that siRNA-mediated Nox4 silencing significantly reduced the mRNA levels of pro-inflammatory genes such as Tnfα, Il1b, and iNOS, confirming the anti-inflammatory effect of Nox4 inhibition in inflammatory macrophages. To further verify whether this anti-inflammatory effect depends on the Nrf2 signaling pathway, we performed rescue experiments using the specific Nrf2 inhibitor ML385. RT-qPCR results showed that ML385 treatment effectively reversed the inhibitory effect of siNox4 on the mRNA expression of pro-inflammatory genes (Tnf, Il1b, and Nos2), functionally validating that Nrf2 activation is required for the anti-inflammatory effect induced by Nox4 inhibition. Recent studies have highlighted the crucial role of the Nox4-Nrf2 redox balance in the pathogenesis of knee osteoarthritis (Li X. et al., 2023). Nox4 has been implicated in generating excessive ROS, potentially by modulating the activation of endogenous antioxidant system (Greatorex et al., 2023). Therefore, we investigated whether silencing Nox4 could inhibit inflammation via modulating Nrf2-related antioxidant system in inflammatory macrophage. Our results revealed that Nrf2 downstream antioxidant genes, such as HO-1 and Cat, were significantly upregulated in the siNox4+LPS group, compared to the LPS group. Meanwhile, Western blot results revealed a significant upregulation of Nrf2 and its downstream antioxidant enzymes, HO-1 and Nqo1, along with a marked downregulation of Keap1 protein expression. Collectively, our findings indicate that silencing Nox4 reduced inflammatory reactions by activating the Nrf2 signaling pathway.

After confirming that knockdown of Nox4 could inhibit inflammatory response by activating the endogenous antioxidant system. We next explored intracellular ROS production after siNox4 treatment in LPS-stimulated macrophages. Immunofluorescence staining showed a significant downregulation of iNOS expression following Nox4 silencing, accompanied by a notable upregulation of Nrf2 expression. Meanwhile, intracellular ROS and ^•^O_2_^−^ were significantly suppressed with siNox4 treatment. To further validate the role of Nox4 in regulating oxidative stress, we performed reverse validation experiments by overexpressing Nox4. As shown in Supplementary Figure S2, Nox4 overexpression significantly increased DCFH-DA fluorescence compared to LPS treatment alone, confirming the pro-oxidative function of Nox4. These observations indicated that Nox4 inhibition could reduce ROS production in LPS-stimulated macrophages and had significant implications for the development of therapeutic strategies targeting Nox4 to reduce inflammation and oxidative stress in TMJ OA.

To gain deeper insights into the anti-inflammatory and antioxidant mechanisms of Nox4 inhibition, we conducted RNA sequencing on LPS-activated macrophages treated with the Nox4 inhibitor GLX351322. Volcano plots and heatmaps clearly demonstrated that GLX351322 had a distinct suppressive effect on gene expression. Consequently, our focus was primarily directed towards the downregulated differentially expressed genes. GO and signaling pathway enrichment revealed strong enrichment in cell cycle-related processes and p53-signaling pathways among the downregulated genes. Prior studies have indicated that p53 exerts its influence by suppressing the expression of Slc7a11, a crucial component of the cystine/glutamate antiporter, which in turn inhibits cystine uptake and renders cells susceptible to ferroptosis (Jiang et al., 2015). We have confirmed the antioxidant effect of Nox4 inhibition in LPS-stimulated macrophages through the activation of the Nrf2 signaling pathway, leading to the transcription of its downstream genes, including Slc7a11, Gclc, Hmox1, Cat, and Gclm. Notably, the Nrf2/SLC7A11 pathway has been reported to inhibit ferroptosis in bone-related diseases (Deng et al., 2024). Slc7a11 functions as a vital subunit of System xc-, an essential antioxidant system primarily responsible for its transport activity. Inhibition of System xc-activity subsequently impairs cystine uptake, disrupting the synthesis of GSH) (Koppula et al., 2021). Additionally, elevated expression of the glutamate-cysteine ligase catalytic subunit (Gclc) stimulates GSH synthesis, resulting in the reduction of ROS (Han et al., 2024). Given these findings, we hypothesized that the inhibition of inflammation in macrophages might occur through the activation of the Nrf2/SLC7A11 pathway, leading to alleviation of ferroptosis. Therefore, we investigated the FPKM values of Nrf2 downstream genes, including Slc7a11, Gclc, Hmox1, Cat, and Gclm, in GLX-treated cells. Our results showed significant upregulation of these genes with GLX treatment. Collectively, our findings suggest that Nox4 inhibition may exert anti-inflammatory and antioxidant effects by activating the endogenous antioxidant system and alleviating ferroptosis. These results provide novel insights into the mechanisms underlying the protective effects of Nox4 inhibition in LPS-activated macrophages.

In inflammatory macrophages, a significant amount of ROS is generated, primarily consisting of oxygen radicals produced by mitochondria (Chen et al., 2022). Excessive ROS can induce lipid peroxidation, which in turn enhances ferroptosis and subsequently leads to cellular damage (Chen et al., 2021). GSEA revealed that oxidative stress and the mitochondrial respiratory chain were significantly downregulated after GLX treatment. Based on these findings, we hypothesized that inhibiting Nox4 could alleviate ferroptosis by protecting against mitochondrial damage and reducing lipid peroxidation. Our results demonstrated that silencing Nox4 notably reduced the mitochondrial membrane potential and inhibited the production of mitochondrial ROS (mtROS). Concurrently, lipid peroxidation (measured by C11-BODIPY) and the level of ferroptosis (detected by FerroOrange probe) were significantly decreased after Nox4 silencing. We further evaluated ferroptosis-related biochemical markers: silencing Nox4 significantly restored GPX4 protein expression, which was markedly reduced upon LPS stimulation; additionally, LPS-induced depletion of intracellular GSH and reduction of the GSH/GSSG ratio were reversed by Nox4 silencing. Reverse validation through Nox4 overexpression further supported its pro-ferroptotic role, as Nox4 overexpression significantly upregulated Acsl4. Meanwhile, mitochondrial complex-related genes were significant downregulated after Nox4 silencing. In summary, these findings underscore the ability of Nox4 inhibition to suppress ROS generation by protecting mitochondria from oxidative stress-induced damage, thereby inhibiting lipid peroxidation and ultimately ameliorating ferroptosis.

Compared with other studies in the same field, the innovation of this study lies not only in its in-depth exploration of the relationship between NOX4 and cellular oxidative stress responses but also in its further elucidation of the potential mechanism by which NOX4 regulates ferroptosis, a novel form of programmed cell death. Moreover, to enhance the clinical relevance and translational value of this research, this study specifically utilized tissue specimens from human TMJOA patients and performed comprehensive transcriptomic analysis using high-throughput RNA sequencing technology, thereby providing more direct and robust evidence at the molecular level.

Of course, this study has certain limitations. The sample size of human TMJOA clinical specimens used for high-throughput sequencing was relatively small (n = 3 per group). We fully acknowledge that such a small sample size is susceptible to individual variation, which is a common challenge in TMJ research due to the limited synovial tissue volume available for collection. To address this concern and support the reliability of our RNA-seq results, we further verified NOX4 expression by immunofluorescence in an expanded clinical cohort of 6 TMJ OA samples versus 6 control samples, which confirmed significant upregulation of NOX4 in inflamed synovium.

In summary, we demonstrate that Nox4 upregulation drives synovial inflammation and ferroptosis in TMJ OA via suppression of the Nrf2 antioxidant pathway. Targeting Nox4 effectively restores redox balance, protects mitochondrial function, and ameliorates disease progression, offering a potential strategy for TMJ OA treatment.

## 5 Conclusion

In conclusion, this study demonstrates that Nox4 is significantly upregulated in the synovium of TMJ OA patients and rats, and its expression correlates with enhanced inflammatory responses and oxidative stress. Mechanistically, Nox4 silencing activates the Nrf2/Keap1 antioxidant pathway, reduces mitochondrial ROS production and lipid peroxidation, and subsequently alleviates ferroptosis in LPS-stimulated macrophages. Collectively, our findings uncover a novel Nox4-Nrf2-ferroptosis axis that drives synovial inflammation in TMJ OA, and position Nox4 as a promising therapeutic target for TMJ OA.

## Data Availability

The RNA-seq and microarray data generated in this study have been depositedin the Figshare repository and are publicly available at https://doi.org/10.6084/m9.figshare.32904437.
