# Supplementary material for: Targeting Nox4 alleviates TMJ osteoarthritis by restoring redox homeostasis and suppressing ferroptosis in macrophages
Source: Front Pharmacol. 2026 Aug 6;17:1854785. doi: 10.3389/fphar.2026.1854785 (PMC13490131; doi:10.3389/fphar.2026.1854785)
Supplement: Supplementary file 1 [file DataSheet1.docx]

**Targeting Nox4 Alleviates TMJ Osteoarthritis by Restoring Redox Homeostasis and Suppressing Ferroptosis in Macrophages**

Yucheng Wang^3§^, Qilin Li^1§^, Weijia Zhang^4§^, Rui Chao^1^, Xuzhuo Chen^1^, Yexin Wang^1^*, Shanyong Zhang^1,2^* ,Jinze Zhen^1,2^*

^1^ Department of Oral Surgery, Shanghai Key Laboratory of Stomatology & Shanghai Research Institute of Stomatology, National Clinical Research Center for Oral Diseases, Shanghai Ninth People’s Hospital, College of Stomatology, Shanghai Jiao Tong University School of Medicine, Shanghai, 200011, China.

^2^ Department of Oral Surgery, Fengcheng Hospital of Fengxian District, Shanghai Ninth People’s Hospital Fengcheng Branch Hospital, Shanghai, China.

^3^ Department of Orthodontics, Qingdao University Affiliated Hospital, Qingdao City, 266000, China.

^4^ Pacific State medical university Russia.

*Corresponding Authors:

[wangyexin1993@163.com;](mailto:lingjie@shsmu.edu.cn;) [zhangshanyong@126.com;](mailto:zhangshanyong@126.com;) [zhenlich@163.com](mailto:zhenlich@163.com)

^§^ Yucheng Wang, Qilin Li and Weijia Zhang contributed equally to this work.

**Supplemental Methods**

**Cell culture**

The RAW 264.7 murine macrophage cell line was kindly donated by the Cell Bank/Stem Cell Repository of the Chinese Academy of Sciences. These cells were cultured in complete α-MEM medium supplemented with 5% FBS, 100 units/mL of penicillin, and streptomycin. The incubation was conducted at 37 ℃ in an incubator with a 5% CO_2_ atmosphere. The cells were seeded once they reached a confluence of 70-80%.

Bone marrow-derived macrophages (BMMs) were prepared according to previously described methods [1]. In brief, primary BMMs were isolated from the long bones of six-week-old male C57/BL6 mice. Subsequently, the isolated cells were cultured in a complete medium of α-MEM (supplemented with 10% heat-inactivated FBS, 100 U/mL penicillin/streptomycin, and 30 ng/mL MCSF). The cell cultures were maintained in a humidified atmosphere with 5% CO_2_ at 37°C.

**siRNA**

To perform cell transfection, seed 4 × 10^5^ RAW 264.7 cells into each well of a 6-well plate containing aMEM medium supplemented with 5% FBS, 100 units/mL of penicillin, and streptomycin, ensuring a cell density of 30-50% at the time of transfection. Initially, 1.25 μl of 20 μM siRNA stock solution was diluted in 30 μl of 1X riboFECT™ CP Buffer. Next, 3 μl of 1X riboFECT™ CP Reagent was added, and the mixture was gently pipetted to ensure homogeneity, followed by incubation at room temperature for 0-15 minutes to form the transfection complex. Subsequently, the riboFECT™ CP transfection complex was added to complete medium without antibiotics. The culture plate was then placed in a 37°C CO_2_ incubator for 48 hours, followed by exposure to LPS stimulation for 24h. The details of four transfection groups including NC, NC+LPS, siNox4, and siNox4+LPS. Each transfection sample was set up with at least 3 replicate wells.

**Lentivirus transfection**

Lenti‑NOX4, Lenti‑sh‑NOX4, and Lenti‑NC were purchased from OBiO (Shanghai, China). Expression construct of mouse NOX4 was subcloned into pLenti‑EF1‑EGFP‑P2A‑Puro‑CMV‑3xFLAG‑WPRE vector. Cells were transduced at 30–50% confluency. After 12 h, more than 95% of the cells were successfully transduced.

**Quantitative PCR analysis**

To evaluate the expression of anti-inflammatory-related genes, RAW 264.7 were seeded in 6-well plates at a density of 4 × 10^5^ cells/well, after 48h siRNA transfection, cells were pre‑treated with or without ML385, followed by stimulation with or without LPS for another 1 day. Total RNA was isolated using Trizol reagent, following the manufacturer's protocol. Subsequently, 0.5 mg of the purified RNA was used to reverse transcription (RT) utilizing the HiScript III 1st Strand cDNA Synthesis Kit (R312-01; Vazyme, China). For quantitative RT-PCR, the ChamQ Universal SYBR qPCR Master Mix (Q711; Vazyme, China) was employed. Expression levels were normalized to GAPDH abundance through application of the 2^^-ΔΔ^CT method. The standard deviation (S.D.) of technical replicates is indicated by error bars. The primer sequences utilized in this study are listed in Table 1.

**Table 1. Sequences of the RT-qPCR.**

| Gene | Forward | Reverse |
| --- | --- | --- |
| Il1β | TCGCAGCAGCACATCAACAAGAG | AGGTCCACGGGAAAGACACAGG |
| iNOS | ACTCAGCCAAGCCCTCACCTAC | TCCAATCTCTGCCTATCCGTCTCG |
| TNF-α | CAGGCGGTGCCTATGTCTC | CGATCACCCCGAAGTTCAGTAG |
| GAPDH | GGTGAAGGTCGGTGTGAACG | GGTGAAGGTCGGTGTGAACG |

**Western blotting**

To evaluate the expression of ferroptosis-related proteins, RAW 264.7 cells were seeded in 6-well plates at a density of 5 × 10^5^ cells/well, after 48h siRNA transfection, cultured with or without LPS for another 1 day. Total protein was extracted using a buffer solution containing protease inhibitors (P8340; Sigma-Aldrich, China) to release the proteins. Following extraction, cellular debris was removed by centrifugation, and the supernatant was collected as the protein sample. The protein concentration was determined using a Bicinchoninic acid (BCA) protein assay kit (23225; Thermo Fisher Scientific, USA). Samples were mixed with BCA reagent, and the absorbance was measured at a specific wavelength to calculate the protein concentration. Subsequently, the protein samples were dissolved in a loading buffer and separated by 10% SDS-PAGE gel electrophoresis. The proteins on the SDS-PAGE gel were then transferred onto a 0.22 μm polyvinylidene fluoride (PVDF) membrane. The membrane was blocked with 5% BSA in 1 × TBST for 1 hour at room temperature. Afterward, it was incubated overnight at 4°C with primary antibodies (GPX4, 1:1000; Gapdh, 1:1000). Following incubation with fluorescent secondary antibodies for 1 hour at room temperature, the blots were visualized using the Odyssey V3.0 image scanner (Li-COR Inc., Lincoln, NE, USA).

**Intracellular ROS detection**

Intracellular levels of ROS were measured employing the DCFH-DA probe (S0033S; Beyotime Biotechnology, China). BMMs were plated and subjected to a 12-hour Lentivirus transfection, followed by culturing with or without LPS. Subsequently, the cells were incubated in serum-free medium containing 10 μM DCFH-DA for 20 minutes at 37°C. After that, the cells were stained with either Hoechst 33342 or DAPI for nuclear fluorescence, then washed 3 times with warm phosphate-buffered saline (PBS) to remove excess dye. Fluorescent images were captured via CLSM, and semi-quantitative assessment of ROS-positive cells was performed using the Image J software.

**Measurement of GSH/GSSG**

The intracellular level of GSH/GSSG was measured using GSH and GSSG Assay Kit (S0053;Beyotime Biotechnology, China), according to the manufacturer’s instructions. Briefly, RAW 264.7 cells were divided into four groups: negative control (NC) group, NC + LPS group, si‑Nox4 group, and si‑Nox4 + LPS group. After corresponding transfection and LPS stimulation treatments, the cells were collected and lysed in a protein-free solution, then frozen and thawed for three repetitively in liquid nitrogen and 37℃. After centrifugation, the supernatant of the samples was extracted for measurement of total GSH. The absorbance was detected at 412 nm, and the standard curve was used to quantify the content. The formula was shown as follow: GSH = Total Glutathione – GSSG × 2.

**Statistical analysis**

The data were analyzed using GraphPad Prism 9 software (GraphPad Software, San Diego, CA, USA) and presented as mean ± SD. Experiments were conducted independently repeated three times. One-way analysis of variance (ANOVA) was employed for comparing multiple experimental groups, with a P-value of < 0.05 considered significant. Significance levels were indicated as follows: * for *P*<0.05, ** for *P*<0.01, *** for *P*<0.001, and NS for not significant.

Fig. S1. Nrf2 inhibitor ML385 rescues the inflammatory response caused by siNOX4 treatment in LPS-activated macrophages. The results show the RT‒qPCR analysis of proinflammatory gene expression in LPS-stimulated macrophages with various treatment groups.

Fig. S2. Overexpression of NOX4 increased the ROS production in inflammatory macrophage. A. Confocal images of LPS-activated macrophages from various treatment groups stained with DCFH-DA. B. Quantitative analysis of DCFH-DA. C. RT‒qPCR analysis of Acsl4.

Fig. S3. Inhibition of NOX4 restored the anti-ferroptosis function of inflammatory macrophage. A. Protein expression level of GPX4. B. Intracellular GSH/GSSG levels in LPS-activated macrophages.

1.	Wang, Y., et al., *AZD8835 inhibits osteoclastogenesis and periodontitis-induced alveolar bone loss in rats.* J Cell Physiol, 2019. **234**(7): p. 10432-10444.
